# Cytokines Genotype-Phenotype Correlation in Nonalcoholic Steatohepatitis

**DOI:** 10.1155/2017/4297206

**Published:** 2017-08-09

**Authors:** Ioana Corina Bocsan, Mircea Vasile Milaciu, Raluca Maria Pop, Stefan Cristian Vesa, Lorena Ciumarnean, Daniela Maria Matei, Anca Dana Buzoianu

**Affiliations:** ^1^Department of Pharmacology, Toxicology and Clinical Pharmacology, Iuliu Haţieganu University of Medicine and Pharmacy, Marinescu str. No.23, Cluj-Napoca, Romania; ^2^4th Department of Internal Medicine, Iuliu Haţieganu University of Medicine and Pharmacy, Cluj-Napoca, Romania; ^3^3rd Department of Internal Medicine, Iuliu Haţieganu University of Medicine and Pharmacy, Cluj-Napoca, Romania

## Abstract

NASH consists in lipid accumulation in hepatocytes that trigger oxidative stress, secretion of proinflammatory cytokines leading to steatohepatitis (NASH). The study aimed to investigate the levels of proinflammatory (TNF-*α* and IL-6) along with anti-inflammatory cytokine IL-10 in patients with NASH and to correlate the cytokines' level with their polymorphism. Sixty-six patients with NASH and 30 healthy volunteers were included in the study. The plasmatic level of IL-6, IL-10, and TNF-*α* were determined by ELISA. IL-10 -1082 G/A, IL-6 -174 G/C, and TNF-*α* -308 G/A polymorphisms were determined using the PCR-RFLP technique. IL-6, TNF-*α*, and CRP levels were significantly higher in patients with NASH. There was a positive correlation between proinflammatory cytokines and a negative correlation between IL-10 and proinflammatory markers. The G allele and GG genotype of IL-6 -174 G/C polymorphism were more frequently noticed in NASH patients. Regarding IL-10 -1082 G/A polymorphism, the AA genotype was correlated with NASH and with a low plasmatic level of IL-10. The A allele in position 308 of the TNF-*α* gene was associated with high level of cytokine. In conclusion, there was an imbalance between pro- and anti-inflammatory cytokines in NASH patients. IL-10 -1082 G/A and TNF-*α* -308 G/A genotypes were correlated with the plasmatic levels of cytokines.

## 1. Introduction

Nonalcoholic fatty liver disease (NAFLD) is a heterogeneous disease, encompassing from simple steatosis to a much more active and progressive disease, namely nonalcoholic steatohepatitis (NASH). NASH represents one of the causes for liver fibrosis, cirrhosis, and subsequently hepatocellular carcinoma [1, 2].

NASH affects 6–35% of people worldwide, with a median prevalence in Europe between 26 and 29% [2], which may increase up to 80% in obese people [3]. NASH is now considered a social problem in Western countries, because it is a disease without a specific treatment, with a rapidly increasing prevalence due to “western lifestyle” (rich diet, lack of exercise, high incidence of obesity) [2, 3].

Lifestyle intervention including a specific diet can be useful in all forms of NAFLD [4]. It is well known that nowadays diet in Western countries is characterized by increased carbohydrates and saturated lipid intake. Dietary fat saturation and type of consumed carbohydrates play a considerable role in modulating plasma cholesterol level and determining the risk for NASH [5]. The response to dietary saturated fat and carbohydrates is characterized by a considerable interindividual variation [6]. Thus, the control of NASH only by diet could be a real challenge. Along with diet, molecular factors that influence lipid accumulation in liver should be considered.

Although the pathogenesis of NASH is not completely understood, a two-step model has been accepted. In the first phase, an initial metabolic disturbance increases accumulation of free fatty acids and de novo lipogenesis, leading to steatosis [2, 7]. Lipid deposition in hepatocytes increases liver sensitivity to injury and inflammation. The second step includes oxidative stress, and induction of proinflammatory cytokines, which triggers necroinflammation leading to the progression of steatohepatitis to end-stage liver diseases [7, 8].

Cytokines play a critical role as mediators of injury, inflammation, fibrosis, and cirrhosis in NASH [9]. Secretion of proinflammatory cytokines, like IL-6 and TNF-*α*, is induced by a variety of stimuli, including organ damage. They stimulate immune response in order to eliminate damage cells. Anti-inflammatory cytokines, like IL-10, are produced to protect against an excessive immune response and to limit organ damage [10]. If the stimulus persists, the inflammation can become chronic. Thus, the balance between pro- and anti-inflammatory cytokines plays a major role in reducing the progression of NASH to cirrhosis [10, 11].

TNF-*α* is a cytokine with a wide spectrum of inflammatory effects. It promotes hepatic inflammation, lipid deposition and peroxidation, activates the Kupffer cells, and hepatocyte apoptosis [12]. TNF-*α* also accelerates the hepatic synthesis of other cytokines and leads to severe inflammatory response, producing liver steatohepatitis and necrosis [13]. IL-6 has a critical role in the acute-phase reaction in liver, but also in chronic situations, being correlated with disease progression [14, 15]. Therefore, it could represent a diagnostic marker in routine practice to detect inflammatory conditions [11]. IL-10 is one of the major anti-inflammatory cytokines, having an important role to counteract the hyperactive immune responses and to protect the body from excessive cell damage. IL-10 modulates the synthesis and activity of proinflammatory cytokines, including IL-6 and TNF-*α*.

It is well known that environmental factors clearly affect the development and progression of NASH. But the diversity of phenotypes in persons with similar metabolic risk factors strongly involves genetic factors [16]. The genes that encode cytokines are highly polymorphic; the presence of a specific polymorphism may influence the severity and the course of diseases and in some situations even the response to anti-inflammatory therapy.

The aim of the study is to analyze the plasmatic profile of IL-6, TNF-*α*, and IL-10 in patients with NASH, which can be considered targets that modulate oxidative stress and inflammation. The second objective is to analyze the polymorphism of genes that encode IL-6, TNF-*α*, and IL-10 and to do a genotype-phenotype correlation.

## 2. Materials and Method

### 2.1. Patients

The study was observational, analytic, transversal, prospective, and a case-control type.

Sixty-six patients with nonalcoholic steatohepatitis, diagnosed after international criteria (1), were included in the study. The mean age of patients with NASH was 49.27 ± 13.29 years and sex ratio M : F = 1 : 1.06. We also included 30 healthy volunteers, mean age 46.3 ± 11.03 years, and sex ratio M : F = 1 : 1.12 as control group.

Subjects were excluded from the study if they had a history of alcohol consumption over 20 g/day or a previous diagnostic of chronic viral B or C hepatitis, liver cirrhosis, an inflammatory or autoimmune disease, or if they were under any type of treatment with impact on hepatic function or anti-inflammatory drugs. We also excluded patients with active or chronic infectious diseases, cancer, chronic renal failure, pregnant women, or aged <18 years.

The study protocol was approved by the Ethics Committee of the University of Medicine and Pharmacy according to principles from the Declaration of Helsinki. All the patients had signed an informed consent before the study began.

### 2.2. Clinical Evaluation

The patients were clinically evaluated in the Department of Internal Medicine from 4th Medical Clinic and Regional Institute of Gastroenterology and Hepatology by Prof. Dr. Octavian Fodor, Cluj-Napoca, from April 2016 to April 2017.

The following demographic data were recorded: age, sex, personal medical history, and family medical history. We noted the presence of cardiovascular or metabolic comorbidities: ischemic heart disease with or without angina, hypertension, diabetes, and gallbladder stones.

The clinical evaluation was performed at the inclusion moment, and anthropometric parameters were recorded: body weight, height, and waist circumference. The body mass index (BMI) was calculated by dividing weight by height squared (kg/m^2^). We also measured blood pressure (BP) at rest, and diagnose of hypertension was established when patients had systolic and/or diastolic BP over 140/90 mmHg. Diabetes type 2 was diagnosed when patients exhibited at least two determinations of blood glucose more than 120 mg/dL a jeun. Diagnose of ischemic heart disease was established according to the presence of angina pectoris and the ECG changes.

### 2.3. Laboratory Investigations

Blood samples were collected in the morning after a 12 h fasting period. The following parameters were determined: triglycerides (TG), total cholesterol (TC), glucose, total bilirubin (TB), alanine aminotransferase (ALT), aspartate aminotransferase (AST), and alkaline phosphatase (AF), using commercial kits for laboratory analyzer Konelab Prime 60i Thermo Scientific.

### 2.4. Plasmatic Level of Inflammatory Mediators

We measured the plasmatic levels of C reactive protein (CRP), IL-6, IL-10, and TNF-*α*. The 5 mL blood samples were collected into pyrogen-free tubes without anticoagulant. The blood samples were centrifuged in the 1st hour, followed by serum separation. The serum was stored at −80°C until the determination was performed.

The inflammatory parameters were performed using the ELISA technique (Quantikinine R&D, USA and Abbexa USA kits). The samples and standard dilutions were assayed according to the manufacturer's instructions.

### 2.5. Cytokines' Gene Polymorphism

Genotyping was performed by collecting 5 ml of peripheral blood in EDTA tubes from each patient. DNA was extracted from peripheral blood leukocytes using a commercially available kit (PureLink® Genomic DNA Mini Kit, Invitrogen). Allele frequencies of IL-6 -174 G/C, TNF-*α* -308 G/A, and IL-10 -1082 G/A gene polymorphisms were determined using the PCR-RFLP method according to previously described protocols [17–19]. PCR reactions were carried out using the Eppendorf thermocycler (Mastercycler Gradient, Eppendorf, Germany). The amplification products were then digested with the appropriate fast restriction endonuclease (Fermentas MBI, Vilnius, Lithuania). The sequences of primers and corresponding restriction endonuclease are presented in Table 1. Digested PCR products were electrophoresed in 2.5% agarose gels containing Midori green.

### 2.6. Statistical Analysis

The statistical analysis was performed using MedCalc Statistical Software version 17.5.5 (MedCalc Software bvba, Ostend, Belgium; http://www.medcalc.org; 2017). Data were labelled as nominal and continuous variables. The nominal variables were characterized as percentages and frequencies. We tested the normal distribution for continuous variables using the Kolmogorov-Smirnov test. We characterized them as mean and standard deviations (for variables with normal distribution) or as median and 25–75 percentiles (for variables with nonnormal distribution). We used the Kruskal–Wallis test and Spearman's rho correlation coefficient for univariate analysis of continuous variables. Allele and genotype frequencies were analyzed using the chi-square test or Fisher's exact test. The level of statistical significance was set at *p* < 0.05.

## 3. Results

There is no differences between the NASH group and control group regarding mean age (49.27 ± 13.29 versus 46.3 ± 11.03, *p* = 0.2) and sex distribution (1 : 1.06 versus 1 : 1.14) (*p* = 0.9).

### 3.1. Clinical and Biological Evaluation of NASH Patients

The anthropometric parameters and laboratory values of patients with NASH are presented in Table 2.

Thirty-seven patients (56.06%) had associated hypertension, while thirty-one patients (47.7%) were diagnosed with ischemic heart disease with (18.5%) or without angina (29.2%). Five patients (7.6%) had associated gallbladder stones. Diabetes mellitus was present in 13 patients (19.7%), while in 15 patients (22%) prediabetes was observed (either impaired fasting glucose or impaired glucose tolerance).

### 3.2. Serum Inflammatory Parameters

The inflammatory parameters in both groups are presented in Table 3.

Inflammatory markers were significantly higher in patients with NASH compared to the control groups for IL-6 (*p* < 0.001), TNF-*α* (*p* < 0.001), and CRP (*p* < 0.001), but not for IL-10 (*p* = 0.9).

In patients with NASH, there was a moderate positive correlation between values of CRP and IL-6 (*r* = 0.302, *p* = 0.014), IL-6 and TNF-*α* (*r* = 0.297, *p* = 0.015), and a strong one between CRP and TNF-*α* (*r* = 0.528, *p* < 0.001). We also noticed a moderate negative correlation between basal levels of IL-10 and IL-6 (*r* = −0.250, *p* = 0.043) and a strong negative correlation between IL-10 and TNF-*α* (*r* = −0.530, *p* < 0.001).

There was a positive correlation between anthropometric parameters and IL-6 and TNF-*α*. Regarding correlation between inflammatory markers and tests that reflect liver function, we observed a positive correlation between IL-6 and TNF-*α* and total bilirubin and between CRP and AST, BT, and glycaemia (Table 4).

IL-6 serum level was higher in patients with NASH and associated hypertension (25.48 versus 36.79, *p* = 0.014). There is no difference regarding the serum level of cytokines in patients with NASH and associated ischemic heart disease, angina, gallbladder stones, diabetes, or prediabetes conditions (*p* = NS).

### 3.3. Distribution of Genotypes

The genotype distribution among the controls and NASH patients for each gene was in the Hardy-Weinberg equilibrium (HWE).

There is a significant difference between patients with NASH and the control group regarding allele distribution for IL-10 and IL-6 (Table 5). For IL-6 -174 G/C gene polymorphism, the G allele was more frequent than the C allele in both groups. In patients with NASH, the GG genotype is the dominant one, while in the control group we noticed heterozygous genotype in 12 persons (40%). (OR = 2.24, 95%CI = 1.19–4.23, *p* = 0.01).

For the IL-10 -1082 G/A polymorphism, the A allele was the most frequent in both groups. The AA genotype was significantly more frequently observed in the NASH group compared to the control group (OR = 0.52, 95%CI = 0.27–0.98, *p* = 0.04). There were no differences regarding AA, AG, or GG genotype distribution between groups for TNF- *α* -308 G/A polymorphism (*p* = 0.96).

There is no correlation between a specific genotype and the sex of the patients for any of the investigated cytokine. For IL-6 -174 G/C gene polymorphism, the CC genotype was more frequently observed in patients that have associated hypertension (*p* = 0.05). There were no other correlations of investigated genotypes with patients' comorbidities.

In patients with NASH, we also correlated the plasmatic level with a genotype (Table 6). In case of the IL-10 gene, the AA genotype in position 1082 is correlated with low plasmatic level of corresponding cytokine, compared to the GA and GG genotypes (*p* = 0.003) (Table 6). The same observation was noticed in case of the TNF-*α* gene; the presence of A allele is correlated with high plasmatic level of cytokine compared to that of G allele (*p* = 0.049). There is no correlation genotype-phenotype in case of IL-6 -174G/C polymorphism (*p* > 0.05), even if in case of patients with the CC genotype a higher mean values of serum IL-6 was noticed.

## 4. Discussions

The present study describes the plasmatic profile and gene polymorphisms for some cytokines, in patients with NASH, in an attempt to define their roles in the pathogenesis and clinical features of the disease.

In the pathogenesis of NASH, the cytokines represent as central mediators which promote injury and inflammation that may finally lead to end-stage liver diseases [9]. The balance between pro- and anti-inflammatory cytokines plays a major role in reducing the progression of NASH to cirrhosis [10, 11].

In the present study, significant high plasmatic levels of IL-6, TNF-*α*, and CRP were found in patients with NASH compared to the control group. This observation demonstrated a systemic inflammation in patients with NASH. Our results are in accordance with already the published data [15, 20, 21]. Zahran et al. [21] showed that TNF-*α* was increased in all patients with NASH, including subgroup of patients with fibrosis, compared to the control one. But Zahran et al. [21] also noticed a reduction of IL-10 in the serum, observation that is not confirmed in the present study. The plasmatic level of IL-10 was similar in NASH and the control groups in our study. Das and Balakrishnan [20] noticed increased levels of IL-6 and TNF-*α*, but no difference of IL-10 levels between the NASH group and healthy volunteers, similar to present data. The same results were also reported by [13], who suggests that fat accumulation in the liver, oxidative stress, and inflammatory cytokines is closely related.

Overproduction of IL-6 and TNF-*α* is correlated with a deficiency of IL-10 synthesis, which is an anti-inflammatory mediator [22]. In this study, a positive correlation between proinflammatory cytokines and CRP as marker of inflammation was shown, and a negative correlation between IL-6, TNF-*α*, and IL-10 levels was found. Similar negative correlations were observed in other studies [12, 21]. In the mentioned studies, the serum IL-10 progressively decreases with the level of steatosis. In our research, the level of IL-10 was similar to the control group, but it was negatively correlated with proinflammatory cytokines. In the present study, we analyzed the cytokine profile in patients with NASH with no evaluation of disease progression or staging the patients according to the degree of fibrosis.

Proinflammatory cytokines were positively correlated with anthropometric parameters (BMI and waist circumference). The same positive correlation between TNF-*α* and BMI was noticed previously [12, 21, 23]. No correlation of IL-10 with BMI and waist circumference was observed in our study, different from those findings by Zahran et al. [21], who observed a negative correlation. These results can be explained by the fact that in his study only obese patients with NASH were included, compared to our study that included only 48.48% obese patients.

There is no correlation between inflammatory markers and AST or ALT, which is different from the published data [12, 21]. In a study by Zahran et al. [21], AST and ALT were increased according to the severity of the liver disease. We may hypothesize that patients included in this study presented different degrees of inflammation and no fibrosis, which may explain the lack of correlation between inflammatory markers and liver function.

Even if it is known that TNF-*α* mediated the de novo fatty acid synthesis in the liver stimulating the expression of lipogenic genes, in the present study there was no correlation of IL-6 or TNF-*α* and total cholesterol or triglycerides, similar to previous reported data [21, 24]. But Zahran et al. [21] has demonstrated a negative correlation of IL-10 with triglycerides, but not with cholesterol level. Uchil et al. [24] concluded that hypertriglyceridemia rather than hypercholesterolemia can be considered a significant risk factor towards progression of NASH.

It was noticed that differences in cytokine secretions (low level or overproduction) are associated with certain allelic variants of mediator genes [25]. IL-10 gene -1082 G/A polymorphism may influence IL-10 serum level; the AA genotype is correlated with lower IL-10 secretion [19]. Regarding IL-6 -174 G/C polymorphism, it was observed that the G allele is associated with an increased inflammatory response [26], even if in the IL-6 gene transcription is involved in several other polymorphisms with different locations [27]. The -174 G/C polymorphism is associated with obesity [28]. C/G polymorphism of the IL-6 gene has been also related to different IL-6 serum plasma values among healthy individuals [27]. The polymorphism of the TNF-*α* gene is associated with an increased expression of cytokines in both in vitro and in vivo studies [29]. The A allele increases the risk of obesity and systolic BP and reduces insulin level in patients with metabolic syndrome [30].

In our study, we noticed a predominance of the G allele in position 174 of the IL-6 gene in NASH patients, but also in the control group. The GG genotype was more frequent in the NASH group compared to the control one, which is different from the study by Cengiz et al. [31], who reported no difference between NASH patients and healthy volunteers. If we compare the frequency of the GG genotype, its expression was higher in our study than that of Cengiz et al. (71.9% versus 60.5%.) [31]. They did not find the CC genotype in their study, while in the present one, the CC genotype was observed in 13.6% of the patients. This could be explained by different population investigated, with other genetic characteristics. On the other hand, the presence of G allele and GG genotype with higher frequencies in the NASH group compared to the control one may sustain the hypothesis of chronic inflammation in patients with NASH and a central role of IL-6 in this process.

In our study, the CC genotype was correlated with associated hypertension, while in the study by Tosic-Dragovic et al. [32] patients with G allele of the IL-6 gene experienced 1.5-fold higher risks for cerebrovascular accident. A meta-analysis [33] revealed that IL-6 -174 G/C polymorphism was not significantly associated with hypertension under all of the genetic models, but the authors concluded that several studies are needed especially in Europeans and Middle-Eastern populations. We did not find any correlation between genotypes and plasmatic level of IL-6, which may confirm the previous observation that genetic polymorphisms in the promoter region influence IL6 transcription through a complex interaction, not by a simple additive mechanism [27].

Even if the A allele of IL-10 -1082 G/A polymorphism was less frequently observed in the NASH group than in the control one, the AA genotype was significantly dominant in the investigated group. The prevalence of the AA genotype in our patients (31.8%) was higher compared to previous reported data in Caucasian population (19.4%) [34], while the GG genotype had the same frequency in healthy volunteers as previous reported data [34, 35]. Based on these observations, we might conclude that the AA genotype is correlated with NASH in our population. There are no other studies that investigate the distribution of IL-10 1082 genotypes in patients with NASH.

The presence of the A allele and AA genotype was also correlated with a low level of IL-10. It is estimated that 50–70% of IL-10 synthesis variation is due to genetic factors [36]. Low level of IL-10 permits a continuous production of some proinflammatory cytokines, like IL-6 and TNF-*α*, which promote the development of chronic inflammation. This result explains the negative correlation of IL-10 level and proinflammatory cytokines observed in the present study.

Regarding TNF-*α* -308 G/A polymorphism, there was no difference of genotype or allele distributions between the NASH patients and control group. Similar results were reported by Hu et al. [37] in Chinese population with NASH. There is no data regarding the distribution of genotypes in Caucasian population with NASH, but it was demonstrated that the A allele and AA genotype represent a risk factor for autoimmune liver diseases due to an increased production of TNF-*α*. The same results were noticed in the present study, where A allele is associated with higher values of plasmatic TNF-*α*, which may increase the risk of chronic inflammation.

There are some limitations of this study. Firstly, a small number of patients were included in the study. Secondly, we did not classify the severity of NASH in the moment of inclusion, so we cannot analyze the correlation genotype-phenotype from this point of view. It might be interesting to investigate the evolution of cytokine overtime and to correlate them with disease progression. Also, the author cannot establish an influence of diet on severity and degree of inflammation in patients with NASH.

The main strength of the paper is that it is the first study presenting to evaluate the IL-10 -1082 G/A genotypes in NASH. Also, it is the first research that correlates the genotypes of the cytokines' genes with their plasmatic levels in NASH.

## 5. Conclusion

Patients with NASH had high plasmatic level of IL-6, TNF-*α*, and CRP, reflecting a systemic inflammation. There was an imbalance of pro- and anti-inflammatory cytokines in patients with NASH. The AA genotype in position 1082 of the IL-10 gene was correlated with NASH in our population. There was a correlation between expressed genotypes of IL-10 -1082 G/A, TNF-*α* 308 G/A, and the level of secreted cytokines in patients with NASH. Proinflammatory cytokines could represent markers to evaluate changes in liver functions and severity of the disease and to predict the risk for progression.

## Figures and Tables

**Table 1:** Primers sequences and enzymes used for the investigated polymorphisms.

Gene polymorphism	Primers	Restriction endonuclease
IL-10 -1082 G/A	Fw 5′-CACTACTAAGGCTTCCTTGGGA-3′	XagI
	Rev 5′-GTGAGCAAACTGAGGCACAGACAT-3′	

TNF-*α* -308 G/A	Fw 5′-TCCCCAAAAGAAATGGAGGCAATA	NcoI
	GGTTTTGAGGGCCAT-3′	
	Rev 5′-GAGACGTCTGCTGGCTGGGTG-3′	

IL-6 -174 G/C	Fw 5′-GCC TCA ATG ACG ACC TAA GC-3′	NlaIII
	Rev 5′-TCA TGG GAA AAT CCC ACA TT-3′	

**Table 2:** Anthropometric and biological parameters in patients with NASH.

Parameters	Median (25–75th percentile)
BW (kg)	84.5 (73.75–97.25)
H (cm)	176.25 (167.5–181.3)
BMI (kg/cm^2^)	29.92 (27.03–32.31)
Waist circumference (cm)	99.5 (89.75–112)
AST (UI/L)	39 (32–51)
ALT (UI/L)	56 (44–80)
AF (UI/L)	210 (152–264.5)
TB (mg/dL)	0.7 (0.5–0.9)
Glycaemia (mg/dL)	100 (89–113)
TC (mg/dL)	193 (169–231)
TG (mg/dL)	129 (89–198)

**Table 3:** Basal inflammatory parameters in patients with NASH and in the control group.

Parameters	Controls (*n* = 30)	NASH (*n* = 66)
	Median (25–75th percentile)	Median (25–75th percentile)
CRP (ng/mL)	234.43 (0–1969.45)	3539.68 (1492.98–5084.31)
IL-6 (pg/mL)	1.28 (0.87–1.87)	3.25 (2.48–4.68)
IL-10 (pg/mL)	6.74 (3.87–7.62)	7.98 (5.38–8.96)
TNF-*α* (pg/mL)	2.32 (1.55–2.92)	16.56 (0–77.78)

Data are expressed as (median; 25–75th percentile); Significance *p* < 0.05.

**Table 4:** Correlation between inflammatory markers and anthropometric and laboratory parameters.

Parameter	Inflammatory markers			
	IL-6	IL-10	TNF-*α*	CRP
BW (kg)	0.258^∗^	0.141	0.100	0.003
H (cm)	0.326^∗^	0.011	0.269^∗^	0.205
BMI (kg/m^2^)	0.274^∗^	−0.007	0.234^∗^	−0.001
Waist circumference (cm)	0.087	0.056	0.191	0.243^∗^
AST (UI/L)	0.023	0.066	0.033	0.228
ALT (UI/L)	0.134	−0.135	0.086	0.381^∗^
AF (UI/L)	0.369^∗^	−0.105	0.256^∗^	0.161
TC (mg/dL)	0.051	0.051	−0.71	0.027
TG (mg/dL)	0.074	0.01	0.017	0.110
Glycaemia (mg/dL)	0.144	−0.004	0.167	0.258^∗^

^∗^Correlation coefficient (*r*) is significant at 0.05 level.

**Table 5:** Distribution of allele and genotypes for the investigated polymorphism.

SNPs	Genotype	NASH	Control	Odds ratio (95%CI)	*p*
IL-6 -174 G/C	G	71.97%	53.3%	2.24 (1.19–4.23)	0.01
	C	19.13%	46.7%		
	GG	57.6%	33.3%		
	GC	28.8%	40%		
	CC	13.6%	26.7%		

IL-10 -1082 G/A	G	49.24%	35%	0.52 (0.27–0.98)	0.04
	A	50.76%	65%		
	GG	30.3%	46.6%		
	GA	37.9%	36.7%		
	AA	31.8%	16.7%		

TNF-*α* -308 G/A	G	87%	86.67%	0.98 (0.40–2.40)	0.96
	A	13%	13.33%		
	GG	78.8%	76.67%		
	GA	16.7%	20%		
	AA	4.5%	3.33%		

**Table 6:** Correlation genotype-phenotype for IL-6, IL-10, and TNF-*α*.

SNPs	Genotype	Plasmatic level	*p*
IL-6 -174 G/C	GG	3.12 (2.36–4.31)	0.07
	GC	3.61 (2.95–5.5)	
	CC	4.02 (2.04–6.65)	

IL-10 -1082 G/A	GG	9.32 (7.51–10.12)	0.0001
	GA	8.64 (7.37–8.91)	
	AA	5.44 (2.25–7.01)	

TNF-*α* -308 G/A	GG	12.48 (0–67.04)	0.049
	GA	24.36 (0–141.10	
	AA	108.72 (20.01–161.10)	
